# Preventing delayed diagnosis of cancer: clinicians’ views on main problems and solutions

**DOI:** 10.7189/jogh.06.020901

**Published:** 2016-12

**Authors:** Lorainne Tudor Car, Nikolaos Papachristou, Catherine Urch, Azeem Majeed, Mona El–Khatib, Paul Aylin, Rifat Atun, Josip Car, Charles Vincent

**Affiliations:** 1 Department of Primary Care and Public Health, School of Public Health, Imperial College London, UK; 2 Imperial College Healthcare NHS Trust, ‎St Mary’s Hospital, London, UK; 3 Department of Global Health and Population & Department of Health Policy and Management, Harvard School of Public Health, Harvard, Boston, USA; 4 Health Services and Outcomes Research Programme, LKCMedicine, Nanyang Technological University, Singapore; 5 Department of Experimental Psychology, Medical Sciences Division, University of Oxford, UK

## Abstract

**Background:**

Delayed diagnosis is a major contributing factor to the UK’s lower cancer survival compared to many European countries. In the UK, there is a significant national variation in early cancer diagnosis. Healthcare providers can offer an insight into local priorities for timely cancer diagnosis. In this study, we aimed to identify the main problems and solutions relating to delay cancer diagnosis according to cancer care clinicians.

**Methods:**

We developed and implemented a new priority–setting approach called PRIORITIZE and invited North West London cancer care clinicians to identify and prioritize main causes for and solutions to delayed diagnosis of cancer care.

**Results:**

Clinicians identified a number of concrete problems and solutions relating to delayed diagnosis of cancer. Raising public awareness, patient education as well as better access to specialist care and diagnostic testing were seen as the highest priorities. The identified suggestions focused mostly on the delays during referrals from primary to secondary care.

**Conclusions:**

Many identified priorities were feasible, affordable and converged around common themes such as public awareness, care continuity and length of consultation. As a timely, proactive and scalable priority–setting approach, PRIORITZE could be implemented as a routine preventative system for determining patient safety issues by frontline staff.

Timely cancer diagnosis improves patient survival and quality of life [1]. Delayed diagnosis is a major contributing factor to lower cancer survival in the UK compared to a number of European countries [2,3]. The outcomes of an 1 in 3 people in the UK, estimated to develop cancer during their lifetime, are significantly affected by a large national variation in timely cancer diagnosis [4,5]. Delayed cancer diagnosis accounts for 5 to 10 000 premature deaths in England and an extra Ł150 million of the NHS spending annually [2,6].

In the UK, primary care providers play an important role in cancer care pathway as the first point of contact for patients. Cancer is mostly diagnosed on presentation to primary care, upon screening as an incidental finding or after an emergency presentation [5]. Patients diagnosed in primary care are referred to specialists for further diagnostic workup and treatment. Diagnostic delays occur because of late patient presentation, problems at the primary (from presentation to referral) or secondary care level (from referral to final diagnosis) and during screening [7].

Delayed diagnosis of cancer has been extensively researched, particularly with the launch of “Evidence for a National Awareness and Early Diagnosis Initiative” (NAEDI) [5]. However, the UK is still faced with significant regional differences in timely cancer diagnosis [8]. A nationwide, consistent implementation of available evidence alone could lead to a better chance of survival in 20 000 people [8]. Regional differences in the UK cancer outcomes call for safety policies informed by a local prioritization of the effective interventions. Although existing research identifies a number of contributing factors to delayed cancer diagnosis, it is unclear which patient safety interventions would have the highest yield and should be given precedence. Clinicians offer unmatched, first–hand insight into the health care service delivery and can help in establishing a consensus on priorities for timely cancer diagnosis [9–11]. Furthermore, clinicians’ engagement is essential for successful implementation of patient safety interventions. The UK’s new national patient safety programme and the recent Institute of Medicine report on improving diagnosis in health care call for the inclusion of clinicians in identifying high–priority areas for patient safety and timely diagnosis. [12,13]. In this study, we aimed to identify cancer care clinicians’ priorities for prevention of delayed diagnosis of cancer in North West London.

## METHODS

We adopted a definition for delayed diagnosis as “a diagnosis that was unintentionally delayed while sufficient information was available earlier” [14].

**Figure 1:**
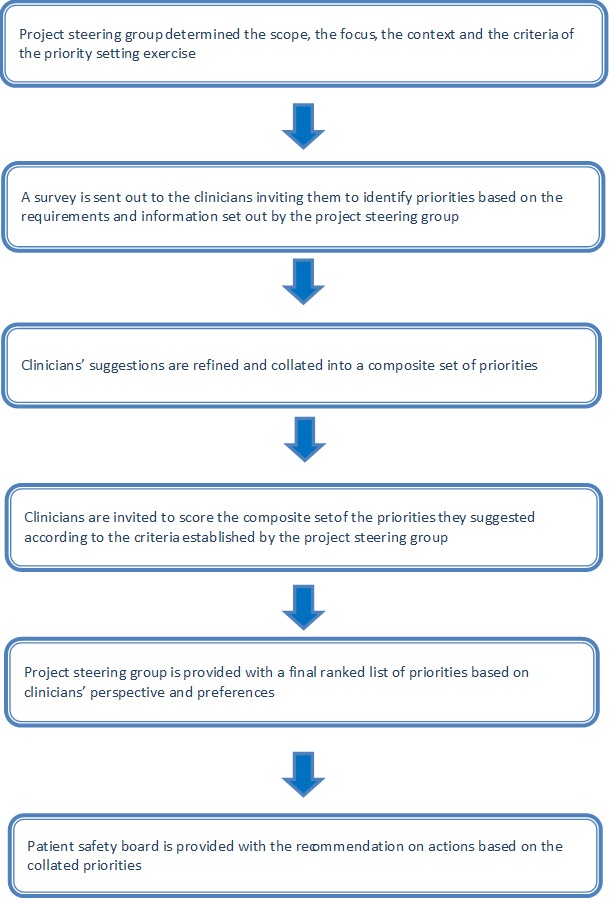
PRIORITIZE methodology flow diagram.

We developed and implemented the PRIORITIZE method, an adaptation of the Child Health and Nutrition Research Initiative (CHNRI) approach, to determine the main problems and solutions relating to delayed diagnosis of cancer [15–17] (**Figure 1**). The CHNRI methodology invites international research experts to nominate priorities for research and has been used extensively to inform policymakers, funding bodies and international organizations. PRIORITIZE is designed to reveal priorities for health care services delivery as seen by clinicians using two complementary angles: problems and solutions. The final output of this approach is presentation of the top priorities categorized according to level of implementation: a) actions for clinicians b) actions for health care organisations and c) actions for health system custodians (**Figure 1**). This study is a service evaluation as well as a quality and safety improvement initiative and therefore did not require ethics or governance approval according to the UK’s Health Research Authority guidance [18,19].

At the study outset, the project steering group (Imperial College Health Partners’ Patient Safety Board) decided to focus on two topics relating to cancer care patient safety: medication safety and delayed diagnosis. This paper describes the findings related to delayed diagnosis of cancer. The project steering group also determined the most pertinent criteria to guide the prioritisation of the collated suggestions, ie, scoring of problems and solutions (**Box 1**).

Box 1 **Scoring criteria for prioritization of collated suggestions**

For problems

**Frequency:** This patient safety threat is common

**Severity:** This patient safety threat leads to high rates of mortality, morbidity and incapacity

**Inequity:** This patient safety threat affects lower socio–economic groups or ethnic minorities more than other groups

**Economic impact:** The consequences of this patient safety threat are costly to the health care system

**Responsiveness to solution:** This incident is amenable to a solution within 5 years

For solutions

**Feasibility**: The implementation of this solution is feasible

**Cost–effectiveness**: This solution is cost–effective

**Potential for saving lives**: This solution would save lives

In the first phase of the study, we developed an open–ended questionnaire for clinicians to identify the main problems and solutions relating to delayed diagnosis in cancer care. The questionnaire was piloted on a smaller sample of primary care physicians and trainees and amended accordingly. The final questionnaire was distributed in both paper–based and online version and disseminated via email lists, snowballing (participants were asked to forward the survey to colleagues), and visits to several general practices in North West London (Text S1 in **Online Supplementary Document(Online Supplementary Document)**). We targeted oncology consultants, general practitioners, trainees, nurses and pharmacists. The collected ideas were examined using content analysis with open coding to categorise the free–text responses. Sufficiently similar suggestions were merged and collated into composite set of priorities.

In the second phase, we created a prioritization matrix consisting of collated priorities and statements outlining prioritization criteria (Box 1 and Text S2 in **Online Supplementary Document(Online Supplementary Document)**). Next, we invited clinicians to categorize the priorities according to the prioritization criteria using four options: score of 1 for ‘Yes – I agree with this statement’, score of 0 for ‘No – I do not agree with this statement’, score of 0.5 for ‘Unsure – I am unsure whether or not I agree’ and no score (blank) for ‘Unaware – I do not feel sufficiently familiar or confident to score this suggestion’ (Text S2 in **Online Supplementary Document(Online Supplementary Document)**). As the scoring process took about an hour to complete, we offered a token payment to the participants in a form of a GBP 50 voucher. From the initial cohort of primary care clinicians, we arbitrarily invited clinicians to perform scoring of the priorities.

**Figure 2:**
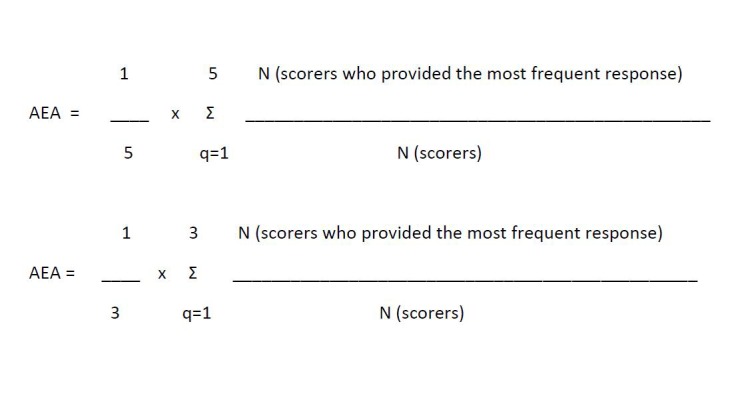
Formula for calculating average experts’ agreement; q is a question that experts are being asked to evaluate competing patient safety threats.

The scores for the suggested priorities were computed as the mean of scores for each of the criteria (ie, five criteria for problems and three for solutions) and ranged from 0 to 100. The Kappa statistic was deemed an inappropriate test to determine inter–rater agreement in this study due to the sample size, the non–standardised categorical nature of data, the option of blank response to some statements and the number of our different criteria used for scoring. Instead, we evaluated the inter–rater agreement using the average expert agreement (AEA). The AEA is the proportion of scorers selecting the mode (the most common score) for each research question. The AEA does not provide information on statistical significance of any differences between scorers, but is pertinent to decision makers as it gives an indication of the degree of agreement between clinicians in terms of priorities. The AEA was calculated using the following formula (**Figure 2**), where q is a question that experts are being asked to evaluate competing patient safety threats (in this case problems leading to delayed diagnosis of cancer), ranging from 1 to 5 for problems and 1 to 3 for solutions.

**Figure 3:**
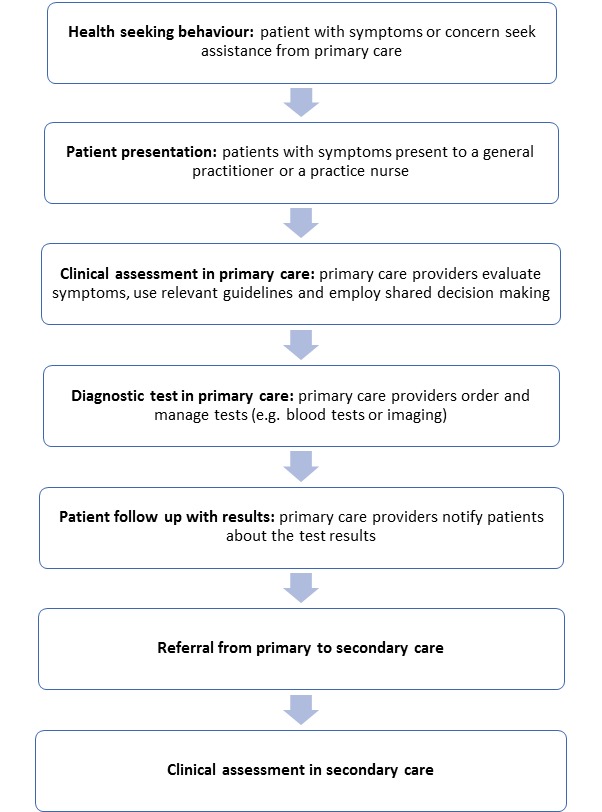
The primary care cancer pathway in the UK (based on “National Patient Safety Agency. Delayed diagnosis of cancer: thematic review, 2010”).

Diagnosis is a multistep process that is influenced by the provider, the patient and the health system [20,21]. In accordance with the patient cancer care pathway in the UK developed by the National Patient Safety Agency (**Figure 3**), we employed an adapted version of a four–dimension model of delays in cancer diagnosis consisting of: 1. Patient delay – from the onset of symptoms to patients’ first presentation, 2. Primary care delay – from the first presentation in primary care to the referral for further care or diagnostic investigation, 3. Referral delay – from the referral for further care or diagnostic investigation to being seen in secondary care, 4. Secondary care delay – from being first seen in secondary care to diagnosis and 5. Screening delay – from being screen to being diagnosed [22,23]. In our analysis, we used an extensively referenced framework categorizing the diagnostic errors into system, cognitive and patient–related factors [14,24]. In addition, solutions were categorized in terms of the type of organizational interventions to decrease the diagnostic errors they addressed, ie, technique, personnel changes, staff educational interventions, structured process changes, technology–based intervention and additional review (Text S3 in **Online Supplementary Document(Online Supplementary Document)**) [25]. To this framework, we added an additional category focused on patient education and empowerment.

## RESULTS

**Figure 4:**
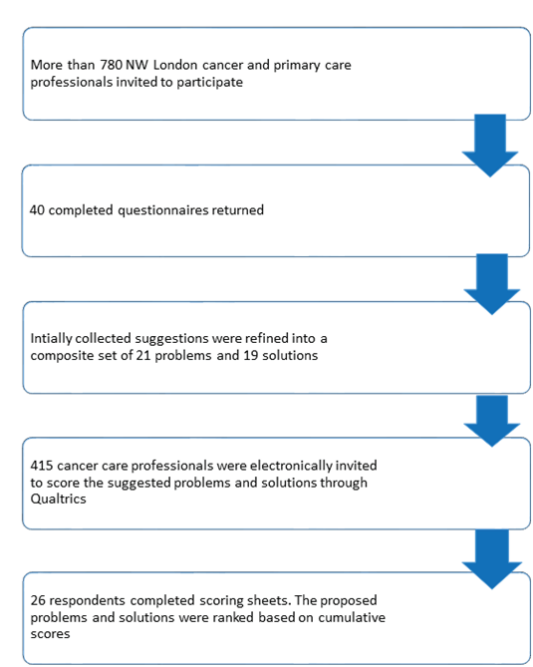
Participants’ flow diagram.

In the first phase, we invited >780 cancer care clinicians and received 40 completed questionnaires, mostly by oncology consultants (n = 15, 37.5%) and trainees (n = 15, 37.5%) (Text S4 in **Online Supplementary Document(Online Supplementary Document)**). 93 problems and 65 solutions relating to delayed diagnosis were thematically merged into a set of 21 distinct problems and 19 solutions. In the second phase, we invited 415 caner care clinicians from the initial cohort to score the composite list of suggestions and received 26 fully completed scoring sheets (**Figure 4**).

The top ranked problems leading to delayed diagnosis of cancer according to clinicians are lack of patient awareness of cancer symptoms, poor continuity of care and delays in referrals to secondary care (**Table 1**). The highest ranked solutions to delayed cancer diagnosis are public awareness campaigns on common symptoms of cancer, better adherence to referral guidelines and improved communication between general and oncology teams in hospitals (**Table 2**).

**Table 1:** Clinicians–identified top ten problems leading to delayed diagnosis of cancer*

Rank	Proposed problem leading to delayed diagnosis of cancer care	Priority score	Type of factor leading to diagnostic error	Type of delay to cancer diagnosis
1	Lack of patient awareness of cancer symptoms mean that they do not attend for advice and investigation in a timely manner	82.5	Patient–related	Patient delay
2	Poor continuity of care for patients leads to symptoms being missed and delayed diagnosis	79	System	Patient delay
3	Delays in referrals eg, GPs not following two–week referral guidelines mean that patients are diagnosed late in the course of the disease	79	System	Referral delay
4	Patients not having a GP mean that they may use other services such as the Emergency Department which are not designed to detect or diagnose cancer and hence present late	78	Patient–related	Patient delay
5	GPs not having enough time mean that they do not take a full history or examine patients fully and miss cancers	78	System	Primary care delay
6	Delays in accessing diagnostics in the community mean that patients wait longer for hospital appointments	76.5	System	Referral delay
7	Patient fears of the diagnosis of cancer mean that they do not seek health advice early in the course of their illness	75	Patient–related	Patient delay
8	Inefficient processes and bureaucracy in hospitals leads to delays in processing referrals and arranging appointments	69.5	System	Referral delay
9	Co–morbidities make it more difficult to diagnose cancer as the symptoms may be confused with those of other existing illnesses	68.5	Cognitive	Primary care delay
10	GPs ignoring alarm symptoms eg, rectal bleeding leads to delays in diagnosis	68	Cognitive	Primary care delay

*The table uses clinicians’ verbatim statements which were only exceptionally reworded for clarity. Clinicians scored problems using the following criteria: frequency, severity, inequity, economic impact and responsiveness to solution (**Box 1**). The scoring options were 1 for “yes” (eg, this problem is common), 0 for “no” (eg, this problem is uncommon), 0.5 for “unsure” (eg, I am unsure if this problem is common) and blank for “unaware” (eg, I do not know if his problem is common). Total priority score is the mean of the scores for each of the five criteria and ranges from 0 to 100. Higher ranked problems received more “Yes” responses for each of the criteria and a higher score.

**Table 2:** Clinicians’ identified top 10 solutions for delayed diagnosis of cancer

Rank	Proposed solution to delayed diagnosis of cancer	Priority score	Categories of Organizational Interventions to Decrease Diagnostic Errors	Type of delay the proposed solution is aimed at
**1**	Encourage public awareness campaigns on common symptoms of cancer to ensure patients present early in the course of their disease	94.1	Patient education and empowerment	Patient delay
2	Improve adherence to referral guidelines to ensure earlier diagnosis	93.3	Educational intervention	Referral delay
3	Improve communication between general and oncology teams in hospitals to improve the standard of care	93.3	Structured–process change	Referral delay
4	Provide prompt feedback to primary care if delayed diagnosis to encourage learning about incidents	90	Educational interventions	Primary care delay
5	Facilitate rapid referrals from primary care to hospitals	89.2	Structured–process change	Referral delay
6	Improve specialist education for doctors and nurses to ensure better standards of care	89.2	Educational interventions	Secondary care delay
7	Improve funding provided to improve services available and provide quicker access to diagnostics and specialists	87.5	Structured–process change	Referral delay
8	Improve access to GPs for patients to ensure earlier diagnosis	85.8	Structured–process change	Patient delay
9	Improve referral and follow up processes to ensure referrals are not lost	85.8	Structured–process change	Referral delay
10	Ensure sufficient staff available to deal with referrals to ensure no delay in processing referrals	84.1	Personnel change	Referral delay

The table uses clinicians’ verbatim statements which were only exceptionally reworded for clarity. Clinicians scored solutions using the following criteria: feasibility, cost–effectiveness and potential for saving lives (**Box 1**). The scoring options were 1 for “yes” (eg, this problem is common), 0 for “no” (eg, this problem is uncommon), 0.5 for “unsure” (eg, I am unsure if this problem is common) and blank for “unaware” (eg, I do not know if his problem is common). Total priority score is the mean of the scores for each of the three criteria and ranges from 0 to 100. Higher ranked solutions received more “Yes” responses for each of the criteria and a higher score.

Most of the top ten problems addressed system–level issues and organization of care (eg, lack of care continuity, short GP consultations leading to inappropriate history taking and examination, delays in ordering and processing referrals and poor access to diagnostic testing) (**Table 1**). Clinicians considered referrals from primary to secondary care as the most liable to the problems leading to delayed cancer diagnosis (Table S5 in **Online Supplementary Document(Online Supplementary Document)**).

Patients’ lack of cancer symptom awareness and the consequent late presentation, poor continuity of care and referral delays were considered top problems leading to delayed diagnosis in cancer care (Table S5 in **Online Supplementary Document(Online Supplementary Document)**). Patients from lower socio–economic groups or ethnic minorities were considered most likely to use other health care services not designed to diagnose cancer. Proposed cognitive–related problems focused mostly on GPs ignoring or overlooking cancer alarm symptoms due to patients’ comorbidities, an unusual presentation and in patients with the low risk of cancer. Clinicians considered diagnostic lapses by midwifes, introduction of lower–threshold referrals and more inclusive screening as least important problems leading to delayed diagnosis of cancer. Errors at the hospital system level, such as referrals being lost or misallocated were also ranked very low.

Overall, proposed solutions focused on the organisational changes with the aim of improving the referral process and the access to diagnostic testing as well as educational interventions aimed primarily at general practitioners (Table S6 in **Online Supplementary Document(Online Supplementary Document)**). The most cost–effective solutions according to the clinicians are public awareness campaigns on common symptoms of cancer to ensure early presentation. Rapid referrals from primary care to hospitals were considered a solution most likely to save lives. The most feasible solution according to clinicians are longer consultations to ensure full examination and history taking. The least important solutions to delayed cancer diagnosis according to clinicians are referring people with a family history of cancer regardless of their symptoms, mandating referral for certain symptoms and tracking patients who do not attend their hospital appointment.

**Figure 5:**
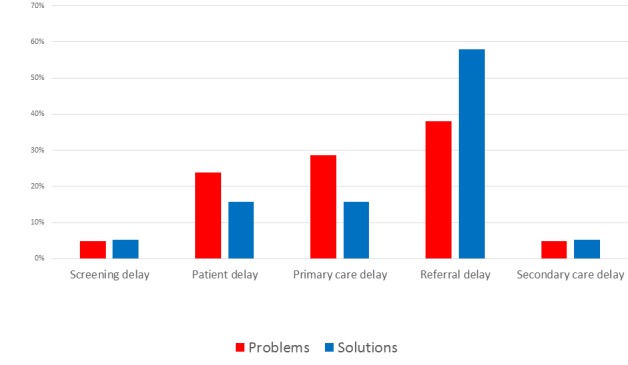
Comparison of problems and solutions related to delayed diagnosis in cancer care in terms of the diagnostic process breakdown point.

The proposed problems and solutions were interrelated as the majority of the identified problems and solutions addressed referrals between primary and secondary care and the top priority in both types of suggestions relates to public awareness of cancer (**Figure 5**). The highest ranked suggestions had the highest AEA, ie, there was a stronger consensus among the clinicians regarding to the top suggestions compared to those ranked lower. The lowest ranked suggestions received a significant number of “Unsure” and “Unaware” answers through scoring (Table S5 in **Online Supplementary Document(Online Supplementary Document)**).

## DISCUSSION

Cancer care clinicians in our study identified a range of priorities for timely diagnosis of cancer in North West London. Lack of cancer symptom recognition among patients, poor continuity of care and complex patient presentation were seen as top problems. Raising public awareness, better patient education and easier access to specialist care and diagnostic testing were seen as top solutions. Referrals from primary to secondary care were considered particularly problematic and likely to cause diagnostic delays. Many suggestions were synergistic or interrelated, and focused on common themes, eg, symptom awareness, care continuity, consultation length etc. This agreement among the identified suggestions reaffirmed the importance of certain priorities in the North West London context and conveys a clear message where action is needed. While all identified problems and solutions are important and revealing, their prioritization can support development of customised, locally–relevant policies in the context of limited health care means.

Clinicians in our study considered patient delays (due to symptom unawareness, late or emergency patient presentation and lack of personal GP) a key problem, more likely to affect lower socio–economic groups. This corresponds to the surveys showing that awareness of cancer warning signs is low, especially among young men and lower socio–economic groups [26,27]. In addition, almost a quarter of all cancer patients in England present as emergencies, often with the later stage of cancers and poorer outcomes [28]. Research shows that poor care continuity, a major problem in our study, hinders timely cancer diagnosis, especially in patients with complex cancer presentation and comorbidities [29,30]. Raising patient awareness through public campaigns, a key solution identified in our study, has been associated with better recognition of symptoms and GP attendance among patients as well with earlier stage lung cancer diagnosis [31,32].

In our study, better adherence to referral guidelines and a quicker access to diagnostic investigations were seen as the top priorities. This is consistent with the recent findings showing that the introduction of “urgent referrals” (seeing a specialist within 2 weeks of presenting to a GP) via National Institute for Health and Care Excellence (NICE) cancer guidelines in 2005 was associated with earlier cancer diagnosis and better patient outcomes [33,34]. And yet, there are still large variations in GP cancer referral rates revealing substantial differences in individual GP thresholds for referring symptomatic patients [35]. Furthermore, instead of mandated “urgent referral”, some patients receive time–consuming diagnostic workup in the primary care setting, leading to later referral for specialist assessment [36]. In our study, clinicians considered rapid referrals to hospitals for certain patients as most likely to save lives which is in fitting with the recent addition of “very urgent referrals” (seeing a specialist within 48 hours of presenting to a GP) to the updated NICE cancer guidelines [37].

### Strengths and limitations

PRIORITIZE is a timely, cost–effective and straightforward answer to calls for engagement of health care staff in patient safety priority–setting [12]. While existing health care provider surveys on delayed diagnosis define priorities according to their frequency [38,39], PRIORITIZE employs several additional relevant and well–defied prioritization criteria such as severity, equity, economic impact and feasibility. Given the regional inequalities in the UK’s cancer care and diagnosis, “one–size–fits–all” approach to development of safety policies and initiatives is unlikely to be successful. PRIORITIZE enables identification of local priorities and implementation of tailored patient safety interventions and policies.

The response rate in our study was low response which nay have affected the generalizability of our findings. However, the number of participants in our study corresponds to those in other priority setting exercises involving health care professionals or employing the CHNRI methodology [40–44]. Furthermore, physician surveys, especially those containing open–ended questions and focusing on sensitive topics, are challenging and in general yield low response rates [45–47]. To boost the response rate, surveys of hospital staff on patient safety in general necessitate leadership engagement, intense campaigning, assurance that the employees’ feedback will be impactful etc. [48]. Surveys in general recruit a self–selected sample and the participants in our study potentially differed from those who did not take part in this study. Although our findings mirror the relevant literature and the participants had the same eligibility criteria by being a cancer care provider in North West London, there may have been other, unmeasured biases. As most of the participants were oncology consultants or hospital–based trainees, this may have also influenced the choice and ranking of priorities.

While our findings are revealing, this approach is at an early stage and could be improved, eg, providing examples to guide specificity of responses (eg, error producing conditions, errors and adverse events), increasing the response rates or enabling longitudinal data collection. PRIORITIZE also provides opportunities for different types of analysis, inclusion of diverse prioritization criteria (eg, urgency, impact, affordability, execution risk, sustainability etc.) and recruitment of both health care professionals and patients.

### Implications for practice and policy

Delayed diagnosis of cancer has been recognized as the key reason for the UK’s lower cancer survival rates. Clinician–identified priorities for a timely cancer diagnosis in our study focused on public awareness, patient education and access to specialist care and diagnostic testing. Using a bottom–up approach, in which clinicians drive change, we collated concrete, locally–relevant and affordable suggestions to inform the health care policy on patient safety. Many suggestions showed agreement underscoring the importance of certain priorities. The Patient Safety Board assembled priorities that were synergistic or inter–related (eg, improving adherence to referral guidelines, improving referral and follow up processes to ensure referrals are not lost, ensuring sufficient staff available to deal with referrals, improving the quality of information in patient referrals) to address them with a focused and concerted effort. Our findings are now being used to guide the Imperial College Health Partners’ work on the Medicines Optimisation in North West London.

Research shows that clinicians often feel excluded from the development of patient safety policies [49], avoid incident reporting due to lack of anonymity or time [50] and are frequently victimised when pointing out safety issues [51]. PRIORITIZE allows transparent, easy reproducible and anonymous voicing of concerns, suggestions and ideas from many health care providers. It triggers staff feedback and involvement, enables evaluation of the organizational culture and the frontline staff views on the locally–relevant patient safety priorities and ultimately aligns the polices with clinicians’ feedback. It also ensures staff calibration, ie, a comparison between the physician’s self–assessment and external overall evaluation of the health care system’s and the organisational safety threats. We propose exploring weather this priority–setting exercise could be included into the annual staff appraisal process to detect clinicians’ perspective on the weaknesses in diagnostic processes in different settings. As a system–wide initiative, PRIORITIZE could increase the awareness of patient safety threats, improve the organisational culture, allow country–wide comparison and implementation of locally tailored–interventions.

## CONCLUSIONS

Clinicians proposed a wide range of implementable, affordable and concrete suggestions for timely cancer diagnosis. The top ranked priorities focused on raising public awareness, patient education as well as better access to specialist care and diagnostic testing. The identified suggestions focused mostly on the delays during referrals from primary to secondary care. While all identified problems and solutions are noteworthy and revealing, their ranking can serve as an aid to policy makers and commissioners of care in prioritization of scarce health care resources. PRIORITIZE is a is highly feasible, informative and scalable priority–setting approach, and merits wider exploration with a view of becoming part of a routine pro–active and preventative system for patient safety assessment.
