# Supplementary material for: Preventing delayed diagnosis of cancer: clinicians’ views on main problems and solutions
Source: J Glob Health. 2016 Nov 24;6(2):020901. doi: 10.7189/jogh.06.020901 (PMC5140077; doi:10.7189/jogh.06.020901)
Supplement: Online Supplementary Document [file jogh-06-020901-s001.pdf]

Online Supplementary Document

Tudor Car et al. Preventing delayed diagnosis of cancer: clinicians' views on main problems and solutions
J Glob Health 2016;6:020901

Text S1. Initial questionnaire on causes of and solutions to delayed diagnosis of cancer

Imperial College London
IMPERIAL COLLEGE HEALTH PARTNERS
WHO Collaborating Centre for Public Health Education and Training

Patient safety in cancer care

Dear *Colleague*,

This survey aims to capture (1) *your* perspective and (2) *your* solutions for incidents that affect the safety of patients with cancer. It has been designed by the Department of Primary Care and Public Health, Imperial College and Imperial College Health Partners to guide patient safety improvement initiatives in NW London and on a wider scale. The results will be used anonymously.

Please specify your role:

Nurse	Specialist Trainee	Consultant	GP	Social Care Professional	Pharmacist	Other (please specify) ____________
○	○	○	○	○	○	○

1. Chemotherapy

A. Please name **3** main chemotherapy-related complications affecting the safety of people in cancer care.

B. How could we reduce or prevent chemotherapy-related complications affecting the safety of patients in cancer care?

2. Delayed Diagnosis

A. Please name **3** main contributors to a delayed diagnosis of cancer.

B. How could we reduce or prevent a delayed diagnosis of cancer?

3. Keeping an eye on care

How could patient safety be better monitored in a GP surgery or a hospital?

4. What's on your mind?

How could we make the care of people with cancer safer?

Thank you very much for your time and effort.

Your contribution will help improve patient safety in cancer care. For more information and to learn more about the outcomes of the study, please contact Dr. Josip Car at josip.car@imperial.ac.uk

Text S2. Scoring questionnaire

Patient safety priorities in cancer care

Dear *Colleague,*

Thank you for participating in the first step of this process where you identified patient safety problems in cancer care and proposed solutions. They are all listed below. In this second and final step, please indicate your agreement or disagreement with all the statements in the table below using the following criteria:

Y(es) : if you agree
N(o) : if you disagree
UnS(ure) : if you are aware of the problem, but unsure about the answer
UnA(ware) : if you are not sufficiently aware of the problem

**Chemotherapy related problems in cancer care**	**This patient safety threat is common**				**This patient safety threat leads to high rates of mortality, morbidity and incapacity**				**This patient safety threat affects more lower socio-economic groups or ethnic minorities**				**The consequences of this patient safety threat are costly to the healthcare system**				**This incident is amenable to a solution within 5 years**			
	Y	N	UnS	UnA	Y	N	UnS	UnA	Y	N	UnS	UnA	Y	N	UnS	UnA	Y	N	UnS	UnA
1. The chemotherapy prescribing system (e.g. ARIA) is separate from the prescribing system for other drugs leading to drug doses and drug interactions being missed	○	○	○	○	○	○	○	○	○	○	○	○	○	○	○	○	○	○	○	○
2. The chemotherapy prescribing system (e.g. ARIA) is difficult to use leading to drug dosing and timing errors	○	○	○	○	○	○	○	○	○	○	○	○	○	○	○	○	○	○	○	○
3. The chemotherapy prescribing system (e.g. ARIA) does not automatically carry forward dose adjustments in regimens leading to inappropriate dosing of chemotherapy drugs	○	○	○	○	○	○	○	○	○	○	○	○	○	○	○	○	○	○	○	○
4. The chemotherapy prescribing system (e.g. ARIA) does not automatically change the dates when chemotherapy is due if one dose is delayed leading to administration of chemotherapy drugs at the wrong time in the cycle	○	○	○	○	○	○	○	○	○	○	○	○	○	○	○	○	○	○	○	○
5. Pharmacists spend significant amounts of time correcting errors on the chemotherapy prescribing system (e.g. ARIA) leading to delays in administration of drugs	○	○	○	○	○	○	○	○	○	○	○	○	○	○	○	○	○	○	○	○
6. Drugs may be stopped for procedures e.g. anticoagulants but not restarted leading to adverse events for patients such as thromboembolic events	○	○	○	○	○	○	○	○	○	○	○	○	○	○	○	○	○	○	○	○
7. Incorrect doses of chemotherapy are administered due to inaccurate calculations of doses	○	○	○	○	○	○	○	○	○	○	○	○	○	○	○	○	○	○	○	○
8. Toxicity or severe allergic reactions from chemotherapy	○	○	○	○	○	○	○	○	○	○	○	○	○	○	○	○	○	○	○	○

9. Interactions between medications are not automatically highlighted meaning that inappropriate drugs may be administered together	○	○	○	○	○	○	○	○	○	○	○	○	○	○	○	○	○	○	○	○

Chemotherapy related problems in cancer care

Chemotherapy related problems in cancer care	This patient safety threat is common				This patient safety threat leads to high rates of mortality, morbidity and incapacity				This patient safety threat affects more lower socio-economic groups or ethnic minorities				The consequences of this patient safety threat are costly to the healthcare system				This incident is amenable to a solution within 5 years			
	Y	N	UnS	UnA	Y	N	UnS	UnA	Y	N	UnS	UnA	Y	N	UnS	UnA	Y	N	UnS	UnA
10. Inappropriate antibiotics are administered for sepsis	○	○	○	○	○	○	○	○	○	○	○	○	○	○	○	○	○	○	○	○
11. Issues such as anaemia, thrombocytopenia or electrolyte disturbance from chemotherapy are not detected due to insufficient monitoring	○	○	○	○	○	○	○	○	○	○	○	○	○	○	○	○	○	○	○	○
12. Too little information on chemotherapy for patients prior to starting treatment meaning that they do not know or recognize signs of complications or serious illness and who and when to contact	○	○	○	○	○	○	○	○	○	○	○	○	○	○	○	○	○	○	○	○
13. Patients attend their GP rather than oncology service for complications from chemotherapy which results in delays in treatment or inappropriate advice or treatments	○	○	○	○	○	○	○	○	○	○	○	○	○	○	○	○	○	○	○	○
14. Patients have difficulty accessing acute oncology services outside of routine hours leading to delayed treatment of side effects or complications with significant negative consequences (e.g. preventable hospitalizations)	○	○	○	○	○	○	○	○	○	○	○	○	○	○	○	○	○	○	○	○
15. Patients are not weighed on each cycle of chemotherapy leading to incorrect doses of chemotherapy on different cycles	○	○	○	○	○	○	○	○	○	○	○	○	○	○	○	○	○	○	○	○
16. Patients do not inform their oncologist of side effects meaning that the chemotherapy dose is not altered and the side effects become worse	○	○	○	○	○	○	○	○	○	○	○	○	○	○	○	○	○	○	○	○
17. Insufficient attention to recognising and managing serious psychological distress or illness due to oncological problem and treatment leads to non-compliance and/or worsening of patient's condition	○	○	○	○	○	○	○	○	○	○	○	○	○	○	○	○	○	○	○	○
18. Inability to obtain information on treatments given in other hospitals or by other healthcare providers e.g. palliative care team mean that the oncology team may administer inappropriate treatments or delay treatment while waiting for the information	○	○	○	○	○	○	○	○	○	○	○	○	○	○	○	○	○	○	○	○
19. Patients with poor understanding of treatments due to language or education difficulties may miss treatments or not understand the importance of reporting side effects leading to worsening of illness	○	○	○	○	○	○	○	○	○	○	○	○	○	○	○	○	○	○	○	○
20. Complications of central access lines inserted for chemotherapy lead to patient morbidity or delayed treatments	○	○	○	○	○	○	○	○	○	○	○	○	○	○	○	○	○	○	○	○

Please add any further ideas or comments that you believe are important and should be included in this study.

Y(es) : if you agree
N(o) : if you disagree
UnS(ure) : if you are aware of the problem, but unsure about the answer
UnA(ware) : if you are not sufficiently aware of the problem

Suggested solutions to chemotherapy related problems in cancer care

Suggested solutions to chemotherapy related problems in cancer care	This solution is cost-effective				The implementation of this solution is feasible				This solution would save lives			
	Y	N	UnS	UnA	Y	N	UnS	UnA	Y	N	UnS	UnA
1. Improve training of staff	○	○	○	○	○	○	○	○	○	○	○	○
2. All patients should receive an appropriate pre-chemotherapy work up	○	○	○	○	○	○	○	○	○	○	○	○

3. Improve the staff:patient ratios	○	○	○	○	○	○	○	○	○	○	○	○
4. Provide individualized information on chemotherapy side effects for patients so they are aware of what to do when unwell	○	○	○	○	○	○	○	○	○	○	○	○
5. Advise patients to check their temperature regularly to detect sepsis earlier	○	○	○	○	○	○	○	○	○	○	○	○
6. Provide information for patients and their carers on what to do when unwell e.g. card with contact numbers	○	○	○	○	○	○	○	○	○	○	○	○
7. Attach the chemotherapy prescription chart to the routine drug chart so drugs are not missed	○	○	○	○	○	○	○	○	○	○	○	○
8. Advise patients to contact hospital early in day if unwell to ensure appropriate staff available	○	○	○	○	○	○	○	○	○	○	○	○
9. Improve symptom control e.g. anti-emetics to reduce side effects and reduce delays in treatments	○	○	○	○	○	○	○	○	○	○	○	○
10. Enable staff to access patient records remotely so that on call staff are fully aware of the patient's history	○	○	○	○	○	○	○	○	○	○	○	○
11. Develop a checklist for clinicians so that important points in the history or tests are not missed	○	○	○	○	○	○	○	○	○	○	○	○
12. Ensure patients have relevant written information for community healthcare professionals to ensure that appropriate treatments are given	○	○	○	○	○	○	○	○	○	○	○	○
13. Improve drug calculation methods to reduce drug dose errors	○	○	○	○	○	○	○	○	○	○	○	○
14. Ensure other medical teams have access to the chemotherapy prescribing system and records in case patient admitted under general medical team to ensure that appropriate monitoring and treatments are provided	○	○	○	○	○	○	○	○	○	○	○	○
15. Improve monitoring of blood tests so that complications such as anaemia or electrolyte abnormalities are not missed	○	○	○	○	○	○	○	○	○	○	○	○
16. Improve communication with pharmacy about drugs and dose adjustments so that delays in drug administration do not occur	○	○	○	○	○	○	○	○	○	○	○	○
17. Improve use of GCSF when appropriate to prevent neutropenia	○	○	○	○	○	○	○	○	○	○	○	○
18. Encourage patients to undertake increased physical activity to improve physical and mental well being	○	○	○	○	○	○	○	○	○	○	○	○
19. Improve acute oncology services e.g. assessment units to fast track patients from the Emergency Department, out of hours phone numbers so that patients are seen quickly by the appropriate specialist	○	○	○	○	○	○	○	○	○	○	○	○
20. Increase use of personalised medicine approaches i.e. targeted therapies to improve outcomes and reduce side effects	○	○	○	○	○	○	○	○	○	○	○	○

**Suggested solutions to chemotherapy related problems in cancer care**	**This solution is cost-effective**				**The implementation of this solution is feasible**				**This solution would save lives**			
	Y	N	UnS	UnA	Y	N	UnS	UnA	Y	N	UnS	UnA
21. Increase the number of clinical nurse specialists to improve patient education and continuity of care	○	○	○	○	○	○	○	○	○	○	○	○
22. Develop chemotherapy prescribing clinics to focus on prescribing and ensure better communication with the chemotherapy nursing team to reduce errors	○	○	○	○	○	○	○	○	○	○	○	○

Please add any further ideas or comments that you believe are important and should be included in this study.

Y(es) : **if you agree**
N(o) : **if you disagree**
UnS(ure) : **if you are aware of the problem, but unsure about the answer**
UnA(ware) : **if you are not sufficiently aware of the problem**

Contributors to delayed diagnosis in cancer care	This patient safety threat is common				This patient safety threat leads to high rates of mortality, morbidity and incapacity				This patient safety threat affects more lower socio-economic groups or ethnic minorities				The consequences of this patient safety threat are costly to the healthcare system				This incident is amenable to a solution within 5 years			
	Y	N	UnS	UnA	Y	N	UnS	UnA	Y	N	UnS	UnA	Y	N	UnS	UnA	Y	N	UnS	UnA
1. Delays in referrals e.g. GPs not following two week referral guidelines mean that patients are diagnosed late in the course of the disease	○	○	○	○	○	○	○	○	○	○	○	○	○	○	○	○	○	○	○	○
2. Referrals get lost in the hospital system meaning that patients are not investigated in a timely manner	○	○	○	○	○	○	○	○	○	○	○	○	○	○	○	○	○	○	○	○
3. Patients present late in the course of the illness due to missed appointments leading to delay in diagnosis	○	○	○	○	○	○	○	○	○	○	○	○	○	○	○	○	○	○	○	○
4. Unusual symptom presentations of cancers mean that patients are not referred for investigation early	○	○	○	○	○	○	○	○	○	○	○	○	○	○	○	○	○	○	○	○
5. Lack of patient awareness of cancer symptoms mean that they do not attend for advice and investigation in a timely manner	○	○	○	○	○	○	○	○	○	○	○	○	○	○	○	○	○	○	○	○
6. Co-morbidities make it more difficult to diagnose cancer as the symptoms may be confused with those of other existing illnesses	○	○	○	○	○	○	○	○	○	○	○	○	○	○	○	○	○	○	○	○
7. Delays in accessing diagnostics in the community mean that patients wait longer for hospital appointments	○	○	○	○	○	○	○	○	○	○	○	○	○	○	○	○	○	○	○	○
8. Patient fears of the diagnosis of cancer mean that they do not seek health advice early in the course of their illness	○	○	○	○	○	○	○	○	○	○	○	○	○	○	○	○	○	○	○	○
9. Patients not having a GP mean that they may use other services such as the Emergency Department which are not designed to detect or diagnose cancer and hence present late	○	○	○	○	○	○	○	○	○	○	○	○	○	○	○	○	○	○	○	○
10. Lack of access to specialist radiology advice leads to delays in treatment plans or inappropriate treatments	○	○	○	○	○	○	○	○	○	○	○	○	○	○	○	○	○	○	○	○
11. GPs ignoring alarm symptoms e.g. rectal bleeding leads to delays in diagnosis	○	○	○	○	○	○	○	○	○	○	○	○	○	○	○	○	○	○	○	○
12. Midwives ignoring concerning symptoms e.g. breast changes during pregnancy leads to a delay in referral and diagnosis	○	○	○	○	○	○	○	○	○	○	○	○	○	○	○	○	○	○	○	○
13. Inappropriate referrals from primary care mean that urgent cases cannot be seen in a timely manner	○	○	○	○	○	○	○	○	○	○	○	○	○	○	○	○	○	○	○	○
14. Screening programs that are too selective mean that some patients are not screened and cancers are missed	○	○	○	○	○	○	○	○	○	○	○	○	○	○	○	○	○	○	○	○
15. GPs not having enough time mean that they do not take a full history or examine patients fully and miss cancers	○	○	○	○	○	○	○	○	○	○	○	○	○	○	○	○	○	○	○	○
16. Inefficient processes and bureaucracy in hospitals leads to delays in processing referrals and arranging appointments	○	○	○	○	○	○	○	○	○	○	○	○	○	○	○	○	○	○	○	○
17. Errors in assigning test priorities in hospital i.e. marking as routine when it should be urgent leads to inappropriate delays in the patient undergoing tests	○	○	○	○	○	○	○	○	○	○	○	○	○	○	○	○	○	○	○	○
18. Low risk patients are not referred for further investigations leading to a delay in diagnosis	○	○	○	○	○	○	○	○	○	○	○	○	○	○	○	○	○	○	○	○

19. Inability to access specialist care leads to a delayed diagnosis	○	○	○	○	○	○	○	○	○	○	○	○	○	○	○	○	○	○	○	○
20. Lack of communication between healthcare professionals leads to referrals not being processed in a timely manner or wrong investigations being ordered	○	○	○	○	○	○	○	○	○	○	○	○	○	○	○	○	○	○	○	○
21. Poor continuity of care for patients leads to symptoms being missed and delayed diagnosis	○	○	○	○	○	○	○	○	○	○	○	○	○	○	○	○	○	○	○	○

Please add any further ideas or comments that you believe are important and should be included in this study.

Y(es) : if you agree
N(o) : if you disagree
UnS(ure) : if you are aware of the problem, but unsure about the answer
UnA(ware) : if you are not sufficiently aware of the problem

Suggested solutions to problems that lead to delayed diagnosis in cancer care	This solution is cost-effective				The implementation of this solution is feasible				This solution would save lives			
	Y	N	UnS	UnA	Y	N	UnS	UnA	Y	N	UnS	UnA
1. Facilitate rapid referrals from primary care to hospitals	○	○	○	○	○	○	○	○	○	○	○	○
2. Encourage primary care to refer more patients and to refer earlier	○	○	○	○	○	○	○	○	○	○	○	○
3. Improve access to GPs for patients to ensure earlier diagnosis	○	○	○	○	○	○	○	○	○	○	○	○
4. Improve referral and follow up processes to ensure referrals are not lost	○	○	○	○	○	○	○	○	○	○	○	○
5. Ensure sufficient staff available to deal with referrals to ensure no delay in processing referrals	○	○	○	○	○	○	○	○	○	○	○	○
6. Encourage public awareness campaigns on common symptoms of cancer to ensure patients present early in the course of their disease	○	○	○	○	○	○	○	○	○	○	○	○
7. Improve specialist education for doctors and nurses to ensure better standards of care	○	○	○	○	○	○	○	○	○	○	○	○
8. Encourage longer consultation times to ensure a full history and examination for presenting symptoms which would lead to an earlier diagnosis	○	○	○	○	○	○	○	○	○	○	○	○
9. Increase screening for cancers to ensure earlier diagnosis	○	○	○	○	○	○	○	○	○	○	○	○
10. Improve access to diagnostics to reduce waiting time for outpatient appointments	○	○	○	○	○	○	○	○	○	○	○	○
11. Improve communication between general and oncology teams in hospitals to improve the standard of care	○	○	○	○	○	○	○	○	○	○	○	○
12. Increase sub-specialisation among cancer specialists to improve the standard of care	○	○	○	○	○	○	○	○	○	○	○	○
13. Do not give clinicians a choice in referral for certain symptoms e.g. mandate that all patients with PR bleeding are referred for a sigmoidoscopy or all breast symptoms are referred to a breast team to ensure that cancers are not missed	○	○	○	○	○	○	○	○	○	○	○	○
14. Refer people with a family history of cancer to oncology even if no symptoms to ensure cancers can be detected earlier	○	○	○	○	○	○	○	○	○	○	○	○
15. Provide prompt feedback to primary care if delayed diagnosis to encourage learning about incidents	○	○	○	○	○	○	○	○	○	○	○	○
16. Improve adherence to referral guidelines to ensure earlier diagnosis	○	○	○	○	○	○	○	○	○	○	○	○
17. If patient does not attend an appointment the hospital should continue to contact them until a response is received to ensure that they do not have a delayed diagnosis	○	○	○	○	○	○	○	○	○	○	○	○

18. Improve the quality of information in patient referrals to enable hospital clinicians to triage referrals better	O	O	O	O	O	O	O	O	O	O	O	O
19. Improve funding provided to improve services available and provide quicker access to diagnostics and specialists	O	O	O	O	O	O	O	O	O	O	O	O

Please add any further ideas or comments that you believe are important and should be included in this study.

Is there anything else for patient safety in cancer care that you would like to share with us?

Thank you very much for your time and effort.

Your contribution will help improve patient safety in cancer care.

For more information and to learn more about the outcomes of the study, please contact Dr. Josip Car at josip.car@imperial.ac.uk

Text S3. Adopted framework used for analysis of classification of solutions to decrease diagnostic errors:*

Technique: Changes in equipment, procedures, and clinical approaches used in clinical practice.

Personnel changes: Introduction of additional or replacement of clinicians

Educational interventions: Educational strategies, training etc.

Structured process changes: Improvements to the existing processes or implementation of additional stages or process in the diagnostic pathway

Technology-based system interventions: Implementation at the system level of tools, such as computer assistive diagnostic aids, decision-support algorithms, text message alerting, and pager alerts

Additional review methods: Introduction of additional independent reviews in the diagnostic pathway

Patient education: Interventions aimed at patient support, improving knowledge of cancer and the relevant health services.

*McDonald KM, Matesic B, Contopoulos-Ioannidis DG, Lonhart J, Schmidt E, Pineda N, et al. Patient safety strategies targeted at diagnostic errors: a systematic review. Ann Intern Med [Internet]. 2013 Mar 5 [cited 2014 Sep 5];158(5 Pt 2):381–9. Available from: http://www.ncbi.nlm.nih.gov/pubmed/23460094

Text S4. Characteristics of the respondents to the initial questionnaire

- Total number of survey respondents: 40
    - Oncology consultant: 15, 37.5%
    - Special Trainee (oncology, general practice, core medical training registrars and foundation doctors): 15, 37.5%
    - Nurse: 6, 15%
    - Cancer research scientists: 2, 5%
    - Pharmacist: 2, 5%

Table S5. Ranking of all (21) delayed diagnosis of cancer problems from clinicians' perspective (AEA range: 0 to 1)

Proposed delayed diagnosis related problems in cancer care	Type of problem	Breakdown point in the diagnostic process	TPS	AEA	Responsiveness to solution	Frequency	Economic impact	Severity	Inequity
Lack of patient awareness of cancer symptoms means that they do not attend for advice and investigation in a timely manner	Patient-related	Patient delay	**1**	**0,77**	**7**	**1**	**5**	**3**	**2**
Poor continuity of care for patients leads to symptoms being missed and delayed diagnosis	System	Patient delay	**2**	**0,75**	**1**	**4**	**10**	**4**	**8**
Delays in referrals e.g. GPs not following two week referral guidelines mean that patients are diagnosed late in the course of the disease	System	Referral delay	**3**	**0,7**	**6**	**3**	**2**	**1**	**6**
GPs not having enough time mean that they do not take a full history or examine patients fully and miss cancers	System	Primary care delay	**4**	**0,72**	**12**	**7**	**1**	**2**	**5**
Patients not having a GP mean that they may use other services such as the Emergency Department which are not designed to detect or diagnose cancer and hence present late	Patient-related	Patient delay	**5**	**0,71**	**10**	**9**	**4**	**10**	**1**
Delays in accessing diagnostics in the community mean that patients wait longer for hospital appointments	System	Referral delay	**6**	**0,64**	**3**	**2**	**3**	**9**	**10**
Patient fears of the diagnosis of cancer mean that they do not seek health advice early in the course of their illness	Patient-related	Patient delay	**7**	**0,69**	**16**	**10**	**7**	**5**	**3**
Inefficient processes and bureaucracy in hospitals leads to delays in processing referrals and arranging appointments	System	Referral delay	**8**	**0,68**	**8**	**8**	**6**	**11**	**15**
Unusual symptom presentations of cancers mean that patients are not referred for investigation early	Cognitive	Primary care delay	**9**	**0,61**	**14**	**14**	**11**	**7**	**4**
Co-morbidities make it more difficult to diagnose cancer as the symptoms may be confused with those of other existing illnesses	Cognitive	Primary care delay	**10**	**0,67**	**20**	**5**	**9**	**8**	**9**

GPs ignoring alarm symptoms e.g. rectal bleeding leads to delays in diagnosis	Cognitive	Primary care delay	**11**	**0,62**	**4**	**18**	**12**	**13**	**11**
Patients present late in the course of the illness due to missed appointments leading to delay in diagnosis	Patient-related	Patient delay	**12**	**0,62**	**17**	**19**	**8**	**6**	**7**
Lack of access to specialist radiology advice leads to delays in treatment plans or inappropriate treatments	System	Referral delay	**13**	**0,61**	**2**	**15**	**16**	**12**	**17**
Inability to access specialist care leads to a delayed diagnosis	System	Referral delay	**14**	**0,59**	**13**	**13**	**17**	**15**	**12**
Lack of communication between healthcare professionals leads to referrals not being processed in a timely manner or wrong investigations being ordered	System	Referral delay	**15**	**0,63**	**5**	**11**	**14**	**18**	**21**
Inappropriate referrals from primary care mean that urgent cases cannot be seen in a timely manner	Cognitive	Referral delay	**16**	**0,58**	**15**	**6**	**13**	**20**	**13**
Referrals get lost in the hospital system meaning that patients are not investigated in a timely manner	System	Referral delay	**17**	**0,56**	**9**	**17**	**15**	**14**	**14**
Errors in assigning test priorities in hospital i.e. marking as routine when it should be urgent leads to inappropriate delays in the patient undergoing tests	System	Secondary care delay	**18**	**0,58**	**11**	**12**	**18**	**16**	**18**
Low risk patients are not referred for further investigations leading to a delay in diagnosis	Cognitive	Primary care delay	**19**	**0,46**	**19**	**20**	**19**	**17**	**16**
Screening programs that are too selective mean that some patients are not screened and cancers are missed	System	Screening delay	**20**	**0,5**	**18**	**16**	**20**	**21**	**19**
Midwives ignoring concerning symptoms e.g. breast changes during pregnancy leads to a delay in referral and diagnosis	Cognitive	Primary care delay	**21**	**0,41**	**21**	**21**	**21**	**19**	**20**

AEA –average expert agreement; TPS – total priority score

Table S6. Ranking of all (19) solutions to delayed diagnosis of cancer from clinicians' perspective (AEA range: 0 to 1)

Proposed solution for delayed diagnosis problems in cancer care	Categories of Organizational Interventions to Decrease Diagnostic Errors	Type of diagnostic delay	TPS	AEA	Cost-effectiveness	Feasibility	Saving Lives
Encourage public awareness campaigns on common symptoms of cancer to ensure patients present early in the course of their disease	Patient education and empowerment	Patient delay	**1**	**0,93**	**3**	**15**	**2**
Improve adherence to referral guidelines to ensure earlier diagnosis	Structured-process change	Referral delay	**2**	**0,90**	**4**	**5**	**10**
Improve communication between general and oncology teams in hospitals to improve the standard of care	Educational intervention	Referral delay	**3**	**0,88**	**1**	**3**	**5**
Provide prompt feedback to primary care if delayed diagnosis to encourage learning about incidents	Educational interventions	Primary care delay	**4**	**0,85**	**2**	**10**	**14**
Facilitate rapid referrals from primary care to hospitals	Structured-process change	Referral delay	**5**	**0,83**	**5**	**16**	**1**
Improve specialist education for doctors and nurses to ensure better standards of care	Educational interventions	Secondary	**6**	**0,82**	**7**	**11**	**7**
Improve funding provided to improve services available and provide quicker access to diagnostics and specialists	Structured-process change	Referral delay	**7**	**0,82**	**12**	**6**	**6**
Improve access to GPs for patients to ensure earlier diagnosis	Structured-process change	Patient delay	**8**	**0,78**	**10**	**7**	**3**
Improve referral and follow up processes to ensure referrals are not lost	Structured-process change	Referral delay	**9**	**0,80**	**6**	**9**	**12**
Ensure sufficient staff available to deal with referrals to ensure no delay in processing referrals	Personnel change	Referral delay	**10**	**0,78**	**11**	**14**	**4**
Improve access to diagnostics to reduce waiting time for outpatient appointments	Structured-process change	Referral delay	**11**	**0,78**	**9**	**19**	**9**
Improve the quality of information in patient referrals to enable hospital clinicians to triage referrals better	Educational intervention	Referral delay	**12**	**0,75**	**8**	**13**	**15**
Increase screening for cancers to ensure earlier diagnosis	Structured-process change	Screening delay	**13**	**0,68**	**13**	**18**	**8**

Encourage longer consultation times to ensure a full history and examination for presenting symptoms which would lead to an earlier diagnosis	Structured-process change	Primary care delay	**14**	**0,67**	**14**	**1**	**13**
Encourage primary care to refer more patients and to refer earlier	Educational interventions	Referral delay	**15**	**0,58**	**15**	**2**	**11**
Increase sub-specialisation among cancer specialists to improve the standard of care	Educational interventions	Primary care delay	**16**	**0,48**	**16**	**4**	**17**
If patient does not attend an appointment the hospital should continue to contact them until a response is received to ensure that they do not have a delayed diagnosis	Structured-process change	Patient delay	**17**	**0,55**	**17**	**17**	**16**
Do not give clinicians a choice in referral for certain symptoms e.g. mandate that all patients with PR bleeding are referred for a sigmoidoscopy or all breast symptoms are referred to a breast team to ensure that cancers are not missed	Structured-process change	Referral delay	**18**	**0,47**	**18**	**8**	**18**
Refer people with a family history of cancer to oncology even if no symptoms to ensure cancers can be detected earlier	Educational intervention	Referral delay	**19**	**0,48**	**19**	**12**	**19**

AEA –average expert agreement; TPS – total priority score
